# Harmful effects of aerosol heavy metals on child health can be increased by their association with organic matter

**DOI:** 10.3389/fpubh.2026.1701563

**Published:** 2026-05-08

**Authors:** Nina Prokopciuk, Lukas Vaidelys, Ulrich Franck, Karolina Sceliokiene, Ugne Zaveckyte, Olga Bielousova, Mindaugas Butikis, Nikolaj Tarasiuk, Arunas Valiulis

**Affiliations:** 1 Clinic of Children's Diseases, Faculty of Medicine, Institute of Clinical Medicine, Vilnius University, Vilnius, Lithuania; 2 Human Ecology Interdisciplinary Research Group, Faculty of Medicine, Institute of Clinical Medicine and Institute of Health Sciences, Vilnius University, Vilnius, Lithuania; 3 Department of Environmental Immunology, Helmholtz Center for Environmental Research—UFZ, Leipzig, Germany; 4 Faculty of Medicine, Vilnius University, Vilnius, Lithuania; 5 Department of Pediatric Gastroenterology and Nutrition, Kharkiv National Medical University, Kharkiv, Ukraine; 6 Department of Public Health, Institute of Health Sciences, Faculty of Medicine, Vilnius University, Vilnius, Lithuania

**Keywords:** aerosol pollution, children, dust, elemental composition, heavy metals, organic matter

## Abstract

**Background:**

It's known that heavy metals are among the most toxic micropollutants. In aerosol particles, heavy metals can be present as chemical compounds and bound to organic matter (ligands). Unlike chemical compounds, the bond between microelements and organic matter is weaker and can be easily destroyed by bacteria releasing biologically active forms of micropollutants.

**Objective:**

To evaluate the impact of the level of association between aerosol heavy metals and organic matter on the incidence of upper respiratory infections in children attending kindergartens.

**Methods:**

Microelemental analysis of aerosols was done using dust samples collected from 22 kindergartens. Concentrations of 11 trace elements were measured by an X-ray fluorescence (ED-XRF) spectrometer. The organic matter content of the dust samples was determined by the extraction method. The organic fraction of each trace element was calculated based on differences in mass and microelemental concentration of the samples before and after extraction (solid fraction). The annual incidence of respiratory infections in each kindergarten was calculated based on medical records data.

**Results:**

A significant correlation was found between indoor concentrations of vanadium (V) and nickel (Ni) and the annual incidence of acute upper respiratory infections in children attending kindergartens. At the same time, the highest detected concentrations of these trace elements were 27.58 ppm (V) and 67.60 ppm (Ni), and did not exceed the permissible age-non-specific concentrations. The association of these microelements with organic matter in dust samples was among the highest for the 11 studied microelements, reaching 94% (V) and 78% (Ni), indicating a potentially large release of biologically active forms of heavy metals.

**Conclusions:**

The concentrations of V and Ni in dust samples collected in kindergartens are related to the annual incidence of upper respiratory infections in preschool children. The association of heavy metals with organic matter in aerosols, which is easily broken down by bacteria in the respiratory tract, is apparently a significant primary source of the biological activity of these microelements in the human body. It can explain the harmful effects of relatively low concentrations of aerosol heavy metals on child health.

## 1 Introduction

In recent decades, the measured mass and number concentrations of aerosol particles have been the preferred metrics for indoor and outdoor aerosol pollution (1–3). They have proven helpful for establishing a relationship with a wide range of health outcomes, including morbidity among children and adults with respiratory diseases (4–6). It was reported that the effects of inhaled aerosols depend on the size of the particles, concentration, duration of exposure, site of deposition in the respiratory tract, as well as their specific chemical composition (7).

Heavy metals (HMs) are components of aerosol particles and are among the most toxic micropollutants, affecting children's health. These effects include intellectual disability, neurocognitive disorders, behavioral problems, cancer, respiratory and cardiovascular diseases (8). Exposure to PM2.5 containing chemical compounds may translocate and induce unbalanced intracellular functions at the genetic and epigenetic levels, leading to mutations, carcinogenesis, and multiple diseases (9). The smallest particles (up to 500 nm) can enter cells due to the mechanisms of endocytosis and pinocytosis. Larger particles are neutralized by phagocytosis (10).

However, the harmful effects on cellular function depend on the microelement composition of the particles absorbed by the cell. Our previous study (11) demonstrated that among low-to-middle concentrations of heavy metals (Pb, W, Sb, Sn, Zr, Zn, Cu, Ni, Mn, Cr, V, and As) measured in dust taken from primary schools, vanadium was one that highly and replicable correlated with the incidence of respiratory infections in children. In aerosol particles, microelements can be present as chemical compounds and bound to organic matter (ligands). We definitely need more studies on the chemical speciation of heavy metals and their impact on mobility and toxicity to human health (5, 12).

Tessier et al. (13) proposed a sequential extraction method to determine the relationships between trace elements and soil components. This method explains the chemical or physical conditions under which the metal will be remobilized (14). Metals associated with a given phase will be released upon dissolution of the host phase (15).

Unlike chemical compounds, the bond between microelements and organic matter is much weaker. Thus, carbonates and oxides, which are broken down by changes in the pH environment or through chemical reactions, the bonds between microelements and organic matter are destroyed by bacteria (16). It is particularly important because the human mouth, trachea, and lungs are not sterile. Bacteria reside in the human respiratory tract (17), where they decompose organic matter and facilitate the release of microelement ions that can penetrate cells.

Consequently, microelements associated with organic matter can be rapidly released into metal ions and become mobile. It is important for assessing the biological activity of trace elements in aerosols.

The idea served as a basis for establishing relationships between microelements and organics through an extraction method (14, 18). Using it to investigate the physicochemical forms of trace elements in aerosols, we hypothesized that the activity of aerosol heavy metals may be modified once they enter the human body.

Our aim was to identify heavy metals in dust as natural aggregates of aerosol pollutants that can impact the incidence of acute respiratory infections in children, and to evaluate the level of association between heavy metals and organic matter as a possible modifier of the harmful effects on children's health.

## 2 Materials and methods

To evaluate the impact of aerosol HMs on the incidence of upper respiratory infections in children attending kindergartens, dust samples were collected from these institutions.

It is known that bacteria easily break down compounds of microelements with organic matter. Consequently, microelements that are mainly associated with organic matter in aerosols can be most biologically active when entering the human body. Therefore, microelements associated with organic matter were assessed using the extraction method. Using the Contamination Factor (CF) and Pollution Load Index (PLI), we compared the calculated concentrations of microelements in kindergarten air with the permissible age-non-specific limits of HMs established for the air of public buildings. The annual incidence of respiratory infections in each kindergarten was calculated based on medical records data.

**Figure 1:**
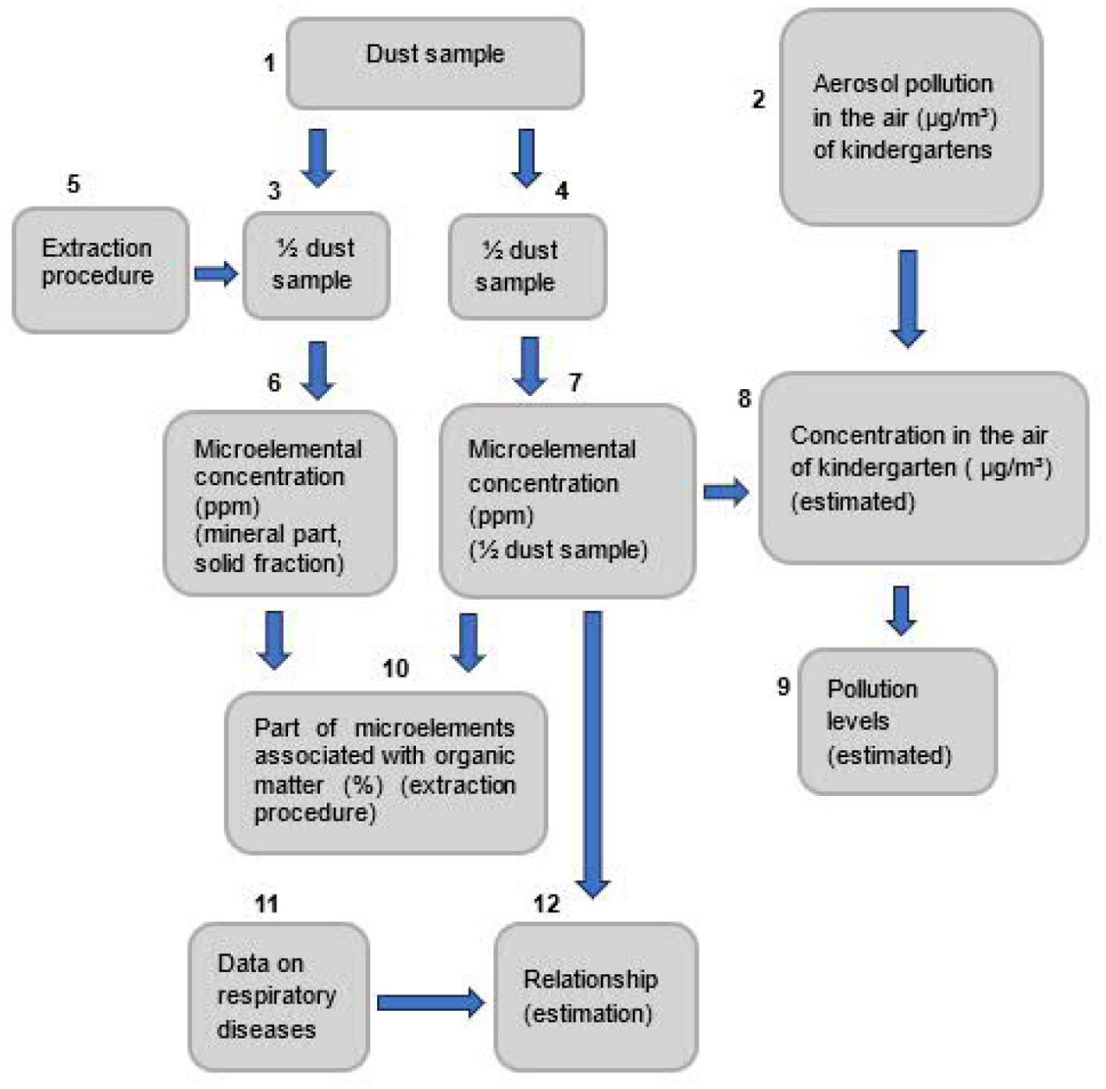
The principal scheme of the study: a dust sample is collected (1); aerosol pollution in the air of kindergartens is measured (5g/m3) (2); dust sample is divided into two equal parts (3, 4); one subsample (3) is ignited (5) and its mineral part is measured for microelemental concentration (ppm) (6); the second subsample (4) is also measured for microelemental concentrations (ppm) (7); HMs concentrations in the air of kindergartens are estimated (5g/m3) (8) and compared with the age-non-specific permissible microelemental pollution levels (9); according to extraction method, microelemental part associated with organic matter is estimated (%) (10); relationship between data on upper respiratory infections (11) and microelemental concentrations in the second subsample (7) is estimated (12).

The principal scheme of our study is presented in Figure 1.

### 2.1 . Collecting dust samples in kindergartens

**Figure 2:**
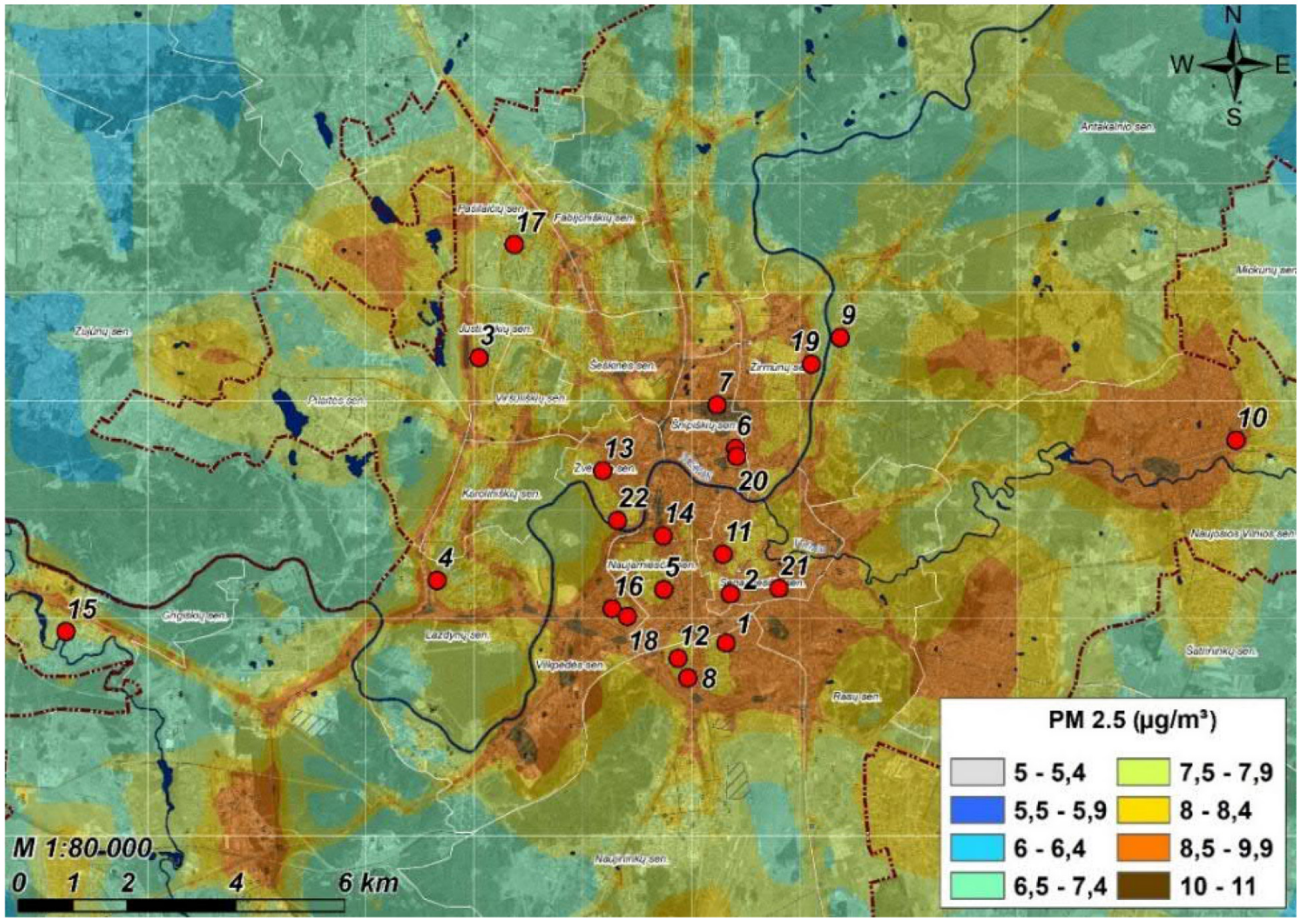
Location of the kindergartens engaged in the research. The scheme of average annual ambient PM_2.5_ concentrations in Vilnius in 2023. Adapted from the public domain “Air Pollution Dispersion Maps” of the Environmental Protection Agency of Lithuania (51), published with permission.

The cross-sectional study was conducted in the Lithuanian capital Vilnius (54°41′17″N, 25°15′8″E) from October 2023 to May 2024. Kindergarten children (aged 3–6 years) were enrolled. Invitations were sent to 38 kindergartens in Vilnius to participate in the study. Ultimately, 22 kindergartens were randomly selected for inclusion (Figure 2).

Dust samples were collected from the majority of children's sleeping rooms and the playrooms in each kindergarten. Dust behind the radiator heaters forms due to aerosol deposits resulting from thermophoretic forces (19). These natural dust aggregates have a purely aerosol origin and are mainly collected against the corners of the rear walls of the radiator heaters. In this case, other dust sources, such as particles brought into the kindergartens by shoes from the street, are not included.

The study was approved by the Vilnius Regional Committee of Biomedical Research Ethics, Protocol No 2024/3-1577-1035.

### 2.2 . Assessing the morbidity of children attending study kindergartens

The annual incidence of doctor-diagnosed acute upper respiratory tract infections (J00–J06) among 3- to 6-year-old children in each kindergarten, involving a total of 3931 children, was calculated based on personal codes and clinical records from health care providers. According to national legislation, personal codes of children and codes of diagnoses based on the Australian Modification of the International Statistical Classification of Diseases and Related Health Problems (ICD-10-AM) were collected and stored by the Lithuanian State Institute of Hygiene.

### 2.3 . Assessing the microelemental composition of dust and aerosol pollutants

A vacuum cleaner with an analytical filter type FPFM (Filtering Polymeric Fibrous Materials) was used for dust collection. Plastic boxes (60 ml, up to 5 g) were tightly filled with collected dust samples. Microelemental analysis of aerosol pollution was carried out using a SPECTRO XEPOS (Spectro Analytical Instruments GmbH, Germany) energy-dispersive X-ray fluorescence (ED-XRF) spectrometer. The concentrations of vanadium (V), chromium (Cr), manganese (Mn), nickel (Ni), copper (Cu), zinc (Zn), arsenic (As), bromine (Br), rubidium (Rb), barium (Ba), and lead (Pb) were measured in dust samples. The measurement time of one sample was 600 s, and the inaccuracy of elemental composition was less than 10%.

The organic matter content of dust samples was determined using the extraction method (13), with minor modifications to isolate the organic matter-bound fraction. A total of 10 mL of 0.02 mol L-1 HNO_3_ and 20 mL of 30% H_2_O_2_ (adjusted to pH 2) were added to the 0.5 g dust sample, and the mixture was heated at 85 °C for 2 h. This treatment was repeated twice to ensure complete oxidation of organic constituents. After cooling, the sample was treated with 20 ml of 3.2 mol L^−1^ CH_3_COONH_4_ prepared in 20% HNO_3_ and heated for an additional 30 min. The resulting suspension was filtered and rinsed with 20 mL of distilled water. Trace-element concentrations were measured in the sample before and after extraction (solid fraction). Organic fractions were calculated from these differences. The microelement composition was analyzed using an X-ray fluorescence (ED-XRF) spectrometer.

An optical particle sizer (OPS, TSI model 3330) was used to determine annual average aerosol particle mass concentrations in the size range of 0.3–10.0 μm (PM_10_). Our previous publication (2) provides a more detailed methodology. Using the concentrations of microelements in the collected dust samples and the average annual indoor PM_10_ levels in kindergartens, the concentrations of microelements (mg/kg) in dust were converted into μg/m3 of air. PM_10_ measurements were conducted in each of 22 study kindergartens.

### 2.4 . Calculating the contamination factor and pollution load index

The contamination factor (CF) is a method used to assess the level of contamination of indoor dust for a particular metal. It is calculated using the following equation (20, 21):


$$\begin{array}{l} CF=\frac{C_{Sample}}{C_{Background}} \end{array}$$ (1)

The CF provides a quantitative measure of the difference in concentration of a specific metal in indoor dust compared to the background level. In this context, the background values for indoor dust are the maximum permissible limits (μg/m3) of pollutants in kindergartens. The maximum permissible limits of contaminants in the air for residential and public buildings are provided in Lithuanian Hygiene Standards (22).

The contamination factor (CF) can be classified as follows: CF < 1 indicates low contamination that does not exceed the maximum permissible limits, while CF >1 indicates contamination that exceeds the maximum permissible limits.

The cumulative pollution load for the total toxic metals at the site is described as the Pollution Load Index (PLI) (20). The PLI for a single site and a zone was calculated from CF (23):


$$\begin{array}{l} PLI\;for\;a\;site={\left(CF_{1}\times {CF_{2}\times \ldots CF}_{n}\right)}^{1/n} \end{array}$$ (2)

$$\begin{array}{l} PLI\;for\;a\;zone={\left(PLI_{Site\;1}\times PLI_{Site\;2}{\times PLI}_{Site\;n}\right)}^{1/n} \end{array}$$ (3)

In this study, the Pollution Load Index (PLI) indicates the total metal pollution at a specific location, particularly at a kindergarten. The PLI is determined by considering the contamination factor (CF) for each element, which shows the level of pollution for each component compared to the maximum permissible limits of that element in the air. Calculating the PLI for an indoor area provides an understanding of the overall pollution load caused by all hazardous metals present. A PLI value of less than 1 means no pollution is detected at the site. A PLI of 1 indicates that the element concentrations in the kindergarten are at the maximum permissible limit. If the PLI exceeds 1, it implies that the air quality at the site has gone beyond the maximum PLC.

### 2.5 . Statistical analysis

This study employed a linear regression model to determine the relationship between respiratory diseases and air pollution. The dependence of the annual incidence of respiratory infections on the microelemental concentration of dust samples can be expressed as a linear function y = a + b·X., where the dependent variable is incidence (%), and the independent variable is microelemental concentration (ppm).

The constant term (intercept) (a) responsible incidence of respiratory infections due to the exposure in the household conditions, the variable term (b·X) proportional to the respective indoor concentration (X) in the kindergarten or school environment, coefficient of proportionality (slope) (b) and dependent variable y—incidence of acute upper respiratory infections (%).

We used Pearson's correlation to evaluate the correlation between the elemental composition of dust samples and the incidence of acute upper respiratory infections in children. A *p*-value of < 0.05 was considered significant. IBM SPSS Statistics 23 was used for statistical analysis.

## 3 Results

### 3.1 . The incidence of acute respiratory infections in children and elemental composition of dust samples

The incidence of upper respiratory infections in each kindergarten ranged from 48.6 to 82.1% in 2024. The highest incidences were observed in kindergartens 4, 6, 10, 15, 16, 18, and 19: 81.1%, 70.8%, 82.1%, 76.1%, 74.6%, 69.8%, and 78.0%, respectively.

**Figure 3:**
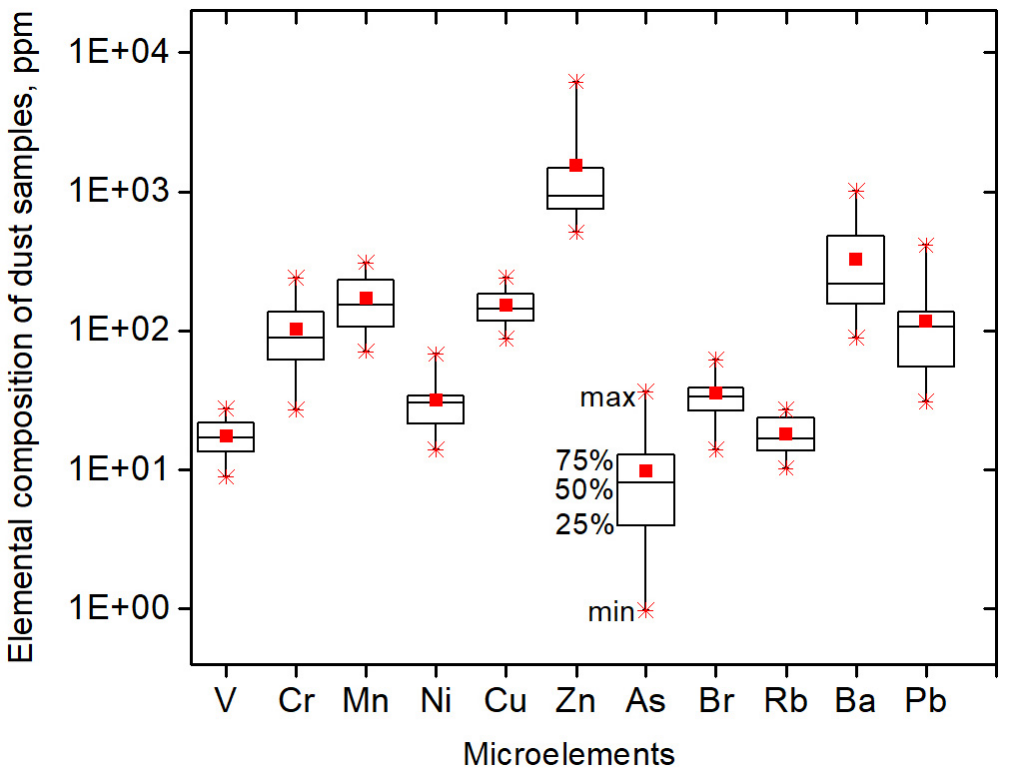
Elemental composition of dust samples in kindergartens (ppm). The square symbols within the boxes indicate the mean values; the star symbols represent the maximum and minimum values; and the boxes represent the 25th, 50th, and 75th percentiles.

Figure 3 presents data on the elemental composition (V, Cr, Mn, Ni, Cu, Zn, As, Br, Rb, Ba, Pb) of dust samples from 22 kindergartens in Vilnius.

Depending on their concentrations, all elements can be divided into several groups. The first group includes elements with up to 70 ppm: V, Ni, As, Br, Rb. The second group consists of elements with concentrations ranging up to 420 ppm, including Cr, Mn, Cu, and Pb. The third group comprises elements Ba and Zn, with concentrations of up to 1,050 ppm and 6,200 ppm, respectively.

**Figure 4:**
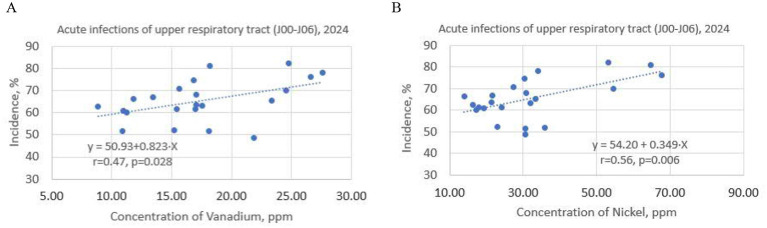
Correlation between vanadium **(A)** and nickel **(B)** concentrations in dust samples and the incidence of acute upper respiratory infections among children in the studied kindergartens.

Regression analysis was performed on all elements in Figure 3. A significant correlation was found between vanadium and nickel concentrations and the incidence of acute upper respiratory infections (J00–J06) in 2024: r = 0.47, *p* = 0.028 for vanadium; r = 0.56, *p* = 0.006 for nickel. The results are summarized in Figures 4A, B.

Linear regression equations were obtained and showed reliable results. The linear regression data for the concentrations of vanadium (ppm) and nickel (ppm) in dust samples, along with the incidence of acute upper respiratory tract infections in 22 kindergartens (annual data), are presented in Table 1.

**Table 1:** Results of linear regression data for vanadium and nickel concentrations in dust samples and incidence of acute upper respiratory infections.

Model	Regression coefficient	Student‘s *t*-test	*p*-value
Constant	50.93	8.02	< 0.0001
Vanadium, ppm	0.823	2.36	0.028
Constant	54.20	13.44	< 0.0001
Nickel, ppm	0.349	3.04	0.006

For vanadium, the F-statistic is 5.58 with a *p*-value of 0.028 and a coefficient of determination (R^2^) of 0.22. For nickel, the F-statistic is 9.23, with a *p*-value of 0.006, and R^2^ of 0.31. Therefore, the *p*-values are < 0.05, indicating the coefficients are reliable. The coefficients of determination (R^2^ = 0.22, R^2^ = 031) indicate that up to 20% of the vanadium data and 21% of the nickel data fit the linear regression model. According to the linear regression equation, a 1-ppm increase in vanadium leads to a 0.77% rise in the incidence of acute upper respiratory infections, while a 1-ppm increase in nickel results in a 0.40% increase in the same incidence. The summary of the results obtained is presented in Figures 4A, B.

The linear correlation indicates that the incidence of acute upper respiratory tract infections is related to at least two air pollution sources. One component depends on school (b·X), and the other on the home environment (a). The coefficient of proportionality (b) ranges from 0.82 for Vanadium to 0.35 for Nickel, while the constant term (a) varies between 50.93 and 54.20, respectively (Figures 4A, B).

### 3.2 . Contamination factor and pollution load index

**Figure 5:**
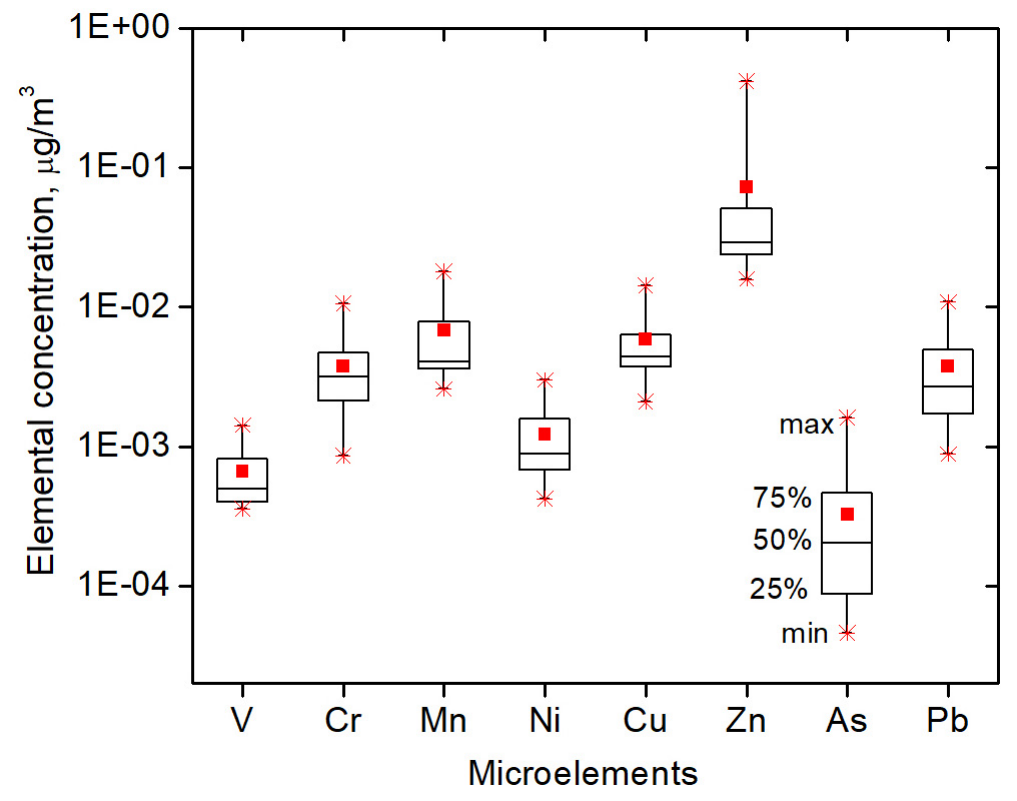
Estimated microelemental concentrations in the air of the studied kindergartens (μg/m^3^). The square symbols within the boxes indicate the mean values; the star symbols represent the maximum and minimum values; and the boxes represent the 25th, 50th, and 75th percentiles of concentration.

Using data on the elemental composition of collected dust in kindergartens and the measured annual mean PM_10_ mass concentrations, the trace element concentrations were calculated in μg/m3 (Figure 5). These values are required to calculate the contamination factor (CF).

The mean values of the computed trace element concentrations ranged from 0.00005 μg/m^3^ of as to 0.4 μg/m^3^ of Zn.

**Figure 6:**
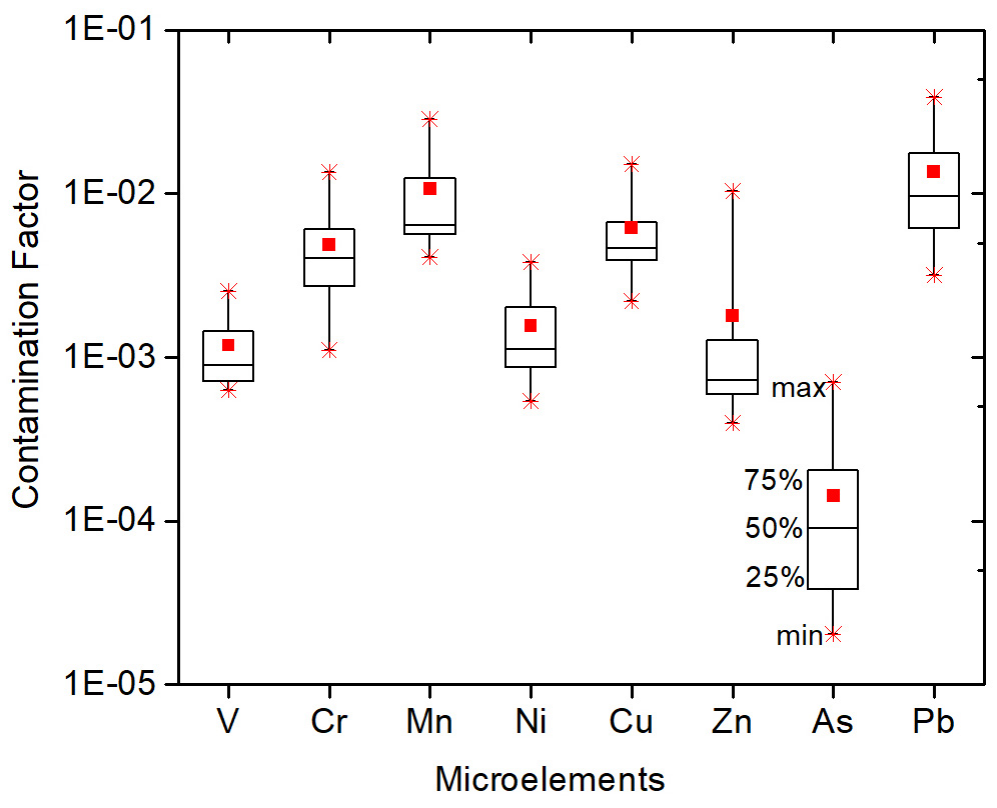
Results of the contamination factor (CF) in the studied kindergartens. The square symbols within the boxes indicate the mean CF; the star symbols represent the maximum and minimum values; and the boxes represent the 25th, 50th, and 75th percentiles of CF.

The results of the contamination factor (CF) (Equation 1) are shown in Figure 6.

All CF values are below 0.04, meaning that no elements exceed the permissible limit value.

**Figure 7:**
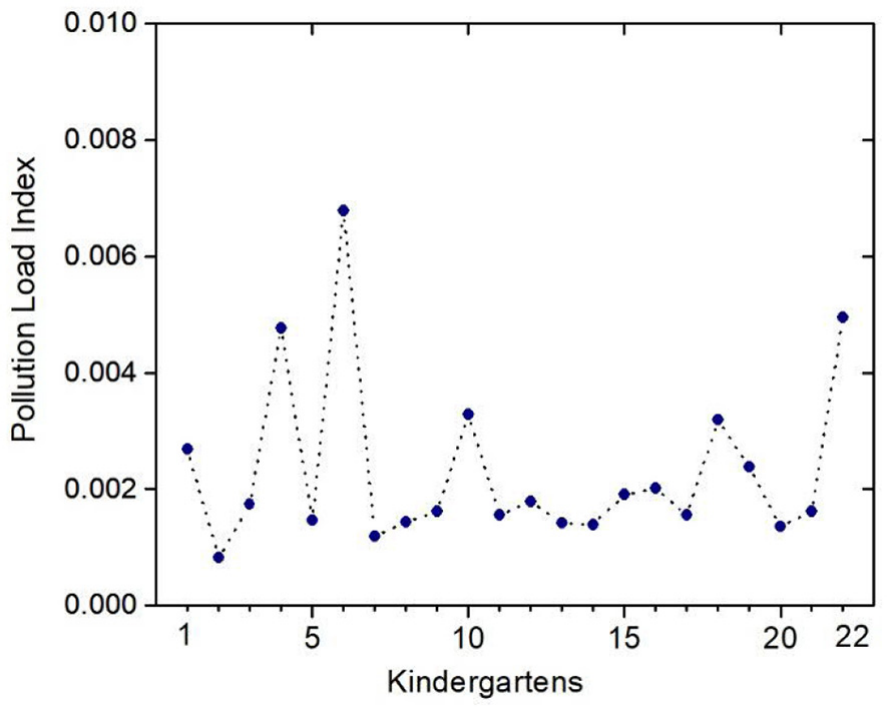
Results of the pollution load index in the studied kindergartens.

The Pollution Load Index (PLI) (Equation 2) in Figure 7 indicates that the total air pollution load of heavy metals, including V, Cr, Mn, Ni, Cu, Zn, As, and Pb in each of the studied kindergartens is minimal (maximum value 0.007) and does not exceed 1. This implies that the concentrations of microelements do not surpass the permissible limit values.

The PLI for all studied kindergartens in Vilnius (PLA for a zone) (Equation 3) equals 0.002.

### 3.3 . Microelement fraction associated with organic matter

**Figure 8:**
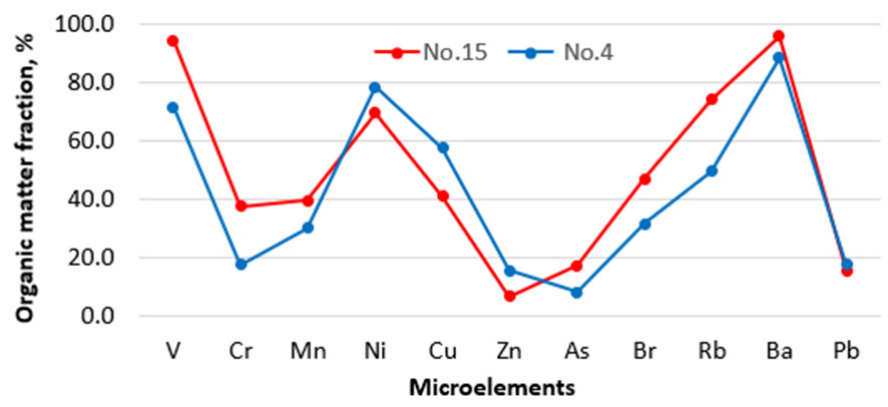
The microelement fractions (%) associated with organic matter in dust samples in four kindergartens (No. 4 and No. 15).

The microelement fractions (%) associated with organic matter are presented in Figure 8.

The most significant fractions (%) of microelements associated with organic matter in dust samples are as follows: V (94%), Ni (78%), Rb (74%), Ba (96%). Their highest concentrations reached were: 26.6 ppm (V), 64.7 ppm (Ni), 11.1 ppm (Rb), and 588 ppm (Ba).

## 4 Discussion

Our research focused on the attempt to explain the proven effects of relatively low concentrations of air pollution on child health (24, 25). It was reported that the importance of individual susceptibility (26, 27), cumulative effects of some pollutants (28), and even non-age-specific permissible levels set by state authorities (22, 29).

In our study, the group of aerosol heavy metals most closely associated with organic matter was identified, including V, Ni, Rb, and Ba. Significant correlations were observed between V, Ni, and the incidence of acute upper respiratory infections among preschool children attending kindergartens, as previously reported by our group in 6- to 11-year-old children attending primary schools (11). A substantial fraction of the V and Ni in dust samples was associated with organic matter (94% and 78%, respectively). This suggests that V and Ni in the human body can be rapidly released as metal ions and become mobile due to weak associations with organic matter, which are rapidly broken down by bacteria (16, 30, 31). Therefore, associations of heavy metals with organic matter could be a significant primary source of biologically active forms. In this regard, chemical compounds of heavy metals are apparently a secondary source of biologically active forms. It seems that chemical compounds are characterized by stronger bonds between trace elements and require chemical reagents to break them down.

Charlson et al. (32) confirm that the lungs of a healthy individual are not sterile but feature a unique community of microorganisms. The most common microorganisms colonizing the bronchial tree in healthy volunteers include bacteria of the genera *Streptococcus, Prevotella, Fusobacterium*, and *Veillonella*. Potentially pathogenic *Haemophilus* and *Neisseria* are less common (33). Among these microorganisms are anaerobes such as *Prevotella spp*. Bacteroidetes (particularly *Prevotella spp*.) are more prevalent in healthy individuals than in patients with bronchial asthma. For example, the healthy nasal cavity is nevertheless enriched with microbial species such as *Corynebacterium spp., Dolosigranulum spp*., and *Moraxella spp* (34). The oropharynx microbiome is primarily composed of *Streptococcus spp*. and exhibits a higher microbial abundance than the nasopharynx (35, 36). While the microbiome of the upper respiratory tract is individual, mainly, fluctuations in bacterial community profiles can be seen across different seasons (winter and summer) and with age (17). The bacteria in the respiratory tract are heterotrophic and obtain energy from organic compounds (16, 30, 31).

Despite a reliable correlation (r = 0.47, *p* = 0.028; r = 0.56, *p* = 0.006), the regression model explains only 22%−31% of the variance in the incidence of acute upper respiratory tract infections linked to V and Ni concentrations in kindergartens. Preschool children spend most of their time at home. Therefore, the home environment plays a more significant role in this context. For schoolchildren aged 6–11 (11), the regression model accounts for up to 68% of the data on the incidence of acute upper respiratory tract infections associated with V concentrations in schools. Applying the linear regression method to the kindergartens and schools, we can infer that the impact of pollution in residential areas on preschoolers is significantly higher than that in the school environment on 6- to 11-year-old children. Thus, the segments (a) cut off from the y-axis of the linear regression line are 50.93 and 54.20 (Figures 4A, B) for the kindergartens and 7.34 for schools, as indicated in our earlier study (11). Another important factor could be the volume of air passing through children's lungs. For example, the weighted average respiratory rate of preschoolers (320 m3/year) is 1.8 times lower than that of schoolchildren (560 m3/year) (37). On the other hand, aerosol particle concentrations in schools are much higher than in kindergartens. Thus, the average PM_2.5_ concentration among schools ranged from 6.8 to 23.0 μg/m^3^ (6), while in kindergartens it varied from 3.3 to 11.4 μg/m^3^ (38).

It is known that metal pollution in residential and commercial areas primarily originates from in-house sources and vehicle emissions (12, 39). Aerosol sampling on filters requires prolonged exposure, and measurements of the microelemental composition, as a rule, are associated with significant uncertainties due to small sample mass. In our study, we sampled dust in areas not reached during routine classroom cleaning (behind the radiator heaters). Sampling a significant amount of dust (up to 5 g) enabled us to determine trace-element concentrations with high accuracy. Also, this amount of collected dust allowed us to analyze the association between a single trace element and organic matter.

In previous studies (40–42), indoor dust does not reflect the composition of aerosol particles because it can originate from various sources. Generally, the dust was collected from floor surfaces using a vacuum cleaner, indicating that it was transported by shoes.

The methodology used by Wilczyńska-Michalik et al. (43) could estimate the parameters of individual large particles (particle size and shape) and their elemental composition. It was found that soot is a common component of aerosol particles collected in this area. In another study from the Upper Silesia region of Poland (44), samples were collected over 5-day periods during the spring and winter seasons. It was evaluated for As, Cd, Cr, Cu, Fe, Mn, Ni, Pb, Sb, Se, and Zn in the studied PM_1_ and Total Suspended Particulate samples. The disadvantage of the method was the inability to perform a more comprehensive analysis and estimate the biological activity of microelements due to the small sample size. Boongla et al. (45), as in our study, used X-ray spectroscopy to examine aerosol particles PM_0.1_ and PM_0.5 − 1_, while industrial microelements were not measured.

Since special attention is given to five elements (V, Ni, As, W, Fe), it is essential to emphasize that the source of all these elements is road traffic (46). Studies in Austria confirm a significant negative correlation between the concentrations of these metals in mosses and the distance from the road. Hu et al. (47) found significant correlations between heavy metal concentrations and traffic; however, only five elements (Cr, Cu, V, Zn, and Pb) were assessed.

Key indicators of tire and brake wear in cars include Cu, Cr, Ba, Zn, and Rb (48). Edible vegetable oils used in frying can also be sources of heavy metals in the air, since all the kindergartens involved in our study had kitchens where fresh food was prepared. The concentrations of Pb, Cd, As, Zn, and Fe in oil samples from Asia were found to be below the suggested legal limits (49). By González-Torres et al. (50), more than 25 heavy metals (with predominant Cd, Pb, Cu, and Fe) were evaluated in 35 different oils (different kinds of sunflower, olive, rapeseed, and corn oils) from 24 countries. However, there is no international legislation regulating the toxicity thresholds of these substances in edible vegetable oils.

In our study, the Contamination Factor and Pollution Load Index do not exceed 1, indicating that the concentrations of microelements (μg/m^3^) remain below the permissible value set by the Lithuanian Hygiene Standards (22). This reinforces the evidence that the concentration of pollutants alone does not explain all effects on the human body, and that other factors, including the degree of association between heavy metals and organic compounds, can modify the harmful effects of pollutants. A significant correlation was observed between Ni, V, and the incidence of acute upper respiratory tract infections, with the predominant fractions of indoor-dust Ni and V associated with organic matter at 74%−89%, respectively.

By sampling dust from long-term accumulation areas of aerosols, we can collect a relatively large mass of samples and improve the accuracy and replicability of the proposed method. This method for evaluating the organic and mineral components of microelements in aerosols enables a careful assessment of the impact of heavy metals on the origin and progression of respiratory disease in children.

Nevertheless, our research has several limitations. Firstly, we did not include data on the home environment, which can be an important modifying factor in this age group of children. Second, the duration of dust natural accumulation in kindergartens was unknown, which prevented us from evaluating single-time data. Third, the respiratory morbidity data were obtained retrospectively from medical records done by medical staff, and it appears that some mild cases of upper respiratory infections may not have been registered. An additional limitation is the small number of kindergartens included in the study. Therefore, further research with a larger number of kindergartens and parallel analysis of the child's home environment should be conducted.

## 5 Conclusions

The concentrations of vanadium and nickel in dust samples collected in kindergartens are related to the annual incidence of upper respiratory infections among preschool children attending these institutions. While the total concentrations of aerosol heavy metals in the studied kindergartens did not exceed the permissible age-non-specific levels, vanadium and nickel compounds with organic matter were as high as 78%−94%. The impact of aerosol heavy metals on child health is related to the amount of microelements associated with organic matter. These associations are the primary source of biologically active forms, resulting from the breakdown of these compounds by bacteria in the respiratory tract of children. This is the first confirmation of the possible impact of heavy metal and organic matter compounds on the initiation/modification of the disease in children, raising awareness of the harmful effects of relatively low concentrations of aerosol heavy metals.

## Data Availability

The original contributions presented in the study are included in the article/supplementary material, further inquiries can be directed to the corresponding author.
